# Supplementary material for: Phage integration alters the respiratory strategy of its host
Source: eLife. 2019 Oct 25;8:e49081. doi: 10.7554/eLife.49081 (PMC6814406; doi:10.7554/eLife.49081)
Supplement: Supplementary file 1. [file elife-49081-supp1.docx]

| **Strain** | **NCBI accession number (strain)** | **Phylogenetic group** | **Isolation source^a^** | ***torT-torS* intergenic distance (bp)** | **Prophage completeness** | **Most similar phage** | **NCBI accession number (phage)** |
| --- | --- | --- | --- | --- | --- | --- | --- |
| NRG 857C | NC_017634 | B2 | Human (Crohn’s disease) | 40,909 | Intact | Sf101 | NC_027398 |
| VR50 | NZ_CP011134 | A | Human urine | 43,597 | Intact | Sf101 | NC_027398 |
| 2011C-4315 | NZ_CP024479 | B1 | Human | 55,336 | Intact | BP-4795 | NC_004813 |
| 2013C-3252 | NZ_CP027442 | B1 | Human | 1,552,843 | Intact | BP-4795 | NC_004813 |
| 2013C-3513 | NZ_CP027555 | B1 | Human | 290,237 | Intact | BP-4795 | NC_004813 |
| 2013C-4187 | NZ_CP027546 | B1 | Human | 766,680 | Intact | BP-4795 | NC_004813 |
| 2013C-4538 | NZ_CP027582 | B1 | Human | 54,007 | Intact | BP-4795 | NC_004813 |
| 2014C-3050 | NZ_CP027472 | B1 | Human | 54,093 | Intact | BP-4795 | NC_004813 |
| 2014C-3051 | NZ_CP027338 | B1 | Human | 2,210,244 | Intact | BP-4795 | NC_004813 |
| 2015C-3163 | NZ_CP027219 | B1 | Human | 95,864 | Intact | BP-4795 | NC_004813 |
| 97-3250 | NZ_CP027599 | B1 | Human | 54,451 | Intact | BP-4795 | NC_004813 |
| CFSAN027343 | NZ_CP037943 | B1 | Human | 55,287 | Intact | BP-4795 | NC_004813 |
| FWSEC0001 | NZ_CP031922 | B1 | Human | 52,669 | Intact | BP-4795 | NC_004813 |
| FWSEC0007 | NZ_CP031908 | B1 | Human | 56,878 | Intact | BP-4795 | NC_004813 |
| FORC_028 | NZ_CP012693 | B1 | Human (food poisoning) | 54,451 | Intact | BP-4795 | NC_004813 |
| FORC_042 | NZ_CP025318 | B1 | Pork | 52,213 | Intact | BP-4795 | NC_004813 |
| PSUO103 | CP014752 | B1 | White-tailed deer (diarrhea) | 55,523 | Intact | BP-4795 | NC_004813 |
| 14EC007 | NZ_CP024131 | A | Human | 48,435 | Intact | CDT-1Φ | NC_009514 |
| E2348/69 | NC_011601 | B2 | Human (diarrhea) | 45,626 | Intact | CDT-1Φ | NC_009514 |
| EPEC1 | NZ_LT903847 | B2 | Human | 41,620 | Intact | CDT-1Φ | NC_009514 |
| C3 | NZ_CP010119 | B1 | Cow | 54,385 | Intact | DE3 | NC_042057 |
| 382634_2f | NZ_CP039403 | B2 | Human | 56,252 | Intact | DE3 | NC_042057 |
| STEC299 | NZ_CP022279 | B2 | Marmot | 48,071 | Intact | GF-2 | NC_026611 |
| 266917_2 | NZ_CP026723 | D | Human | 40,029 | Intact | HK620 | NC_002730 |
| AR_0017 | NZ_CP024886 | B2 | Not stated | 42,171 | Intact | HK620 | NC_002730 |
| KSC64 | NZ_CP018840 | B1 | Pig | 16,119 | Questionable | SEN34 | NC_028699 |
| WAT | NZ_CP012380 | B1 | Pond water | 42,598 | Intact | SEN34 | NC_028699 |
| ECONIH5 | NZ_CP026202 | A | Wastewater | 56,872 | Intact | SEN34 | NC_028699 |
| LF82 | NC_011993 | B2 | Human (Crohn’s disease) | 38,795 | Intact | Sf6 | NC_005344 |
| 214-4 | NZ_CP025840 | A | Human (diarrhea) | 26,441 | Intact | Sf6 | NC_005344 |
| 09-00049 | NZ_CP015228 | B1 | Lettuce | 38,863 | Incomplete | Sf6 | NC_005344 |
| M6 | NZ_CP010186 | B1 | Mouse | 40,155 | Intact | Sf6 | NC_005344 |
| M9 | NZ_CP010196 | B1 | Mouse | 40,155 | Intact | Sf6 | NC_005344 |
| CE10 | NC_017646 | F | Human cerebrospinal fluid (meningitis) | 42,731 | Intact | YYZ-2008 | NC_011356 |
| 13E0767 | NZ_CP020107 | B1 | Cow | 49,427 | Questionable | λ | NC_001416 |
| C7 | NZ_CP010240 | B1 | Cow | 2,092,997 | Intact | λ | NC_001416 |
| RM8352 | NZ_CP028110 | B1 | Creek sediment | 188,739 | Intact | λ | NC_001416 |
| RM14715 | NZ_CP027104 | B2 | Dog | 47,030 | Intact | λ | NC_001416 |
| 16-9255 | NZ_CP022407 | B1 | Flour | 189,841 | Intact | λ | NC_001416 |
| 2014C-3599 | NZ_CP027435 | B1 | Human | 188,698 | Intact | λ | NC_001416 |
| 2014C-3655 | NZ_CP027351 | B1 | Human | 184,560 | Intact | λ | NC_001416 |
| 2014C-4423 | NZ_CP027454 | B1 | Human | 186,166 | Intact | λ | NC_001416 |
| 2015C-3107 | NZ_CP027317 | B1 | Human | 186,607 | Intact | λ | NC_001416 |
| B7A | NZ_CP005998 | B1 | Human | 53,865 | Intact | λ | NC_001416 |
| FWSEC0006 | NZ_CP031910 | B1 | Human | 187,430 | Intact | λ | NC_001416 |

^a^Strain isolated from stool unless otherwise indicated. If disease or clinical sign appears in the NCBI record, it is given in parentheses.
