# Supplementary material for: Phage integration alters the respiratory strategy of its host
Source: eLife. 2019 Oct 25;8:e49081. doi: 10.7554/eLife.49081 (PMC6814406; doi:10.7554/eLife.49081)
Supplement: Supplementary file 2. [file elife-49081-supp2.pdf]

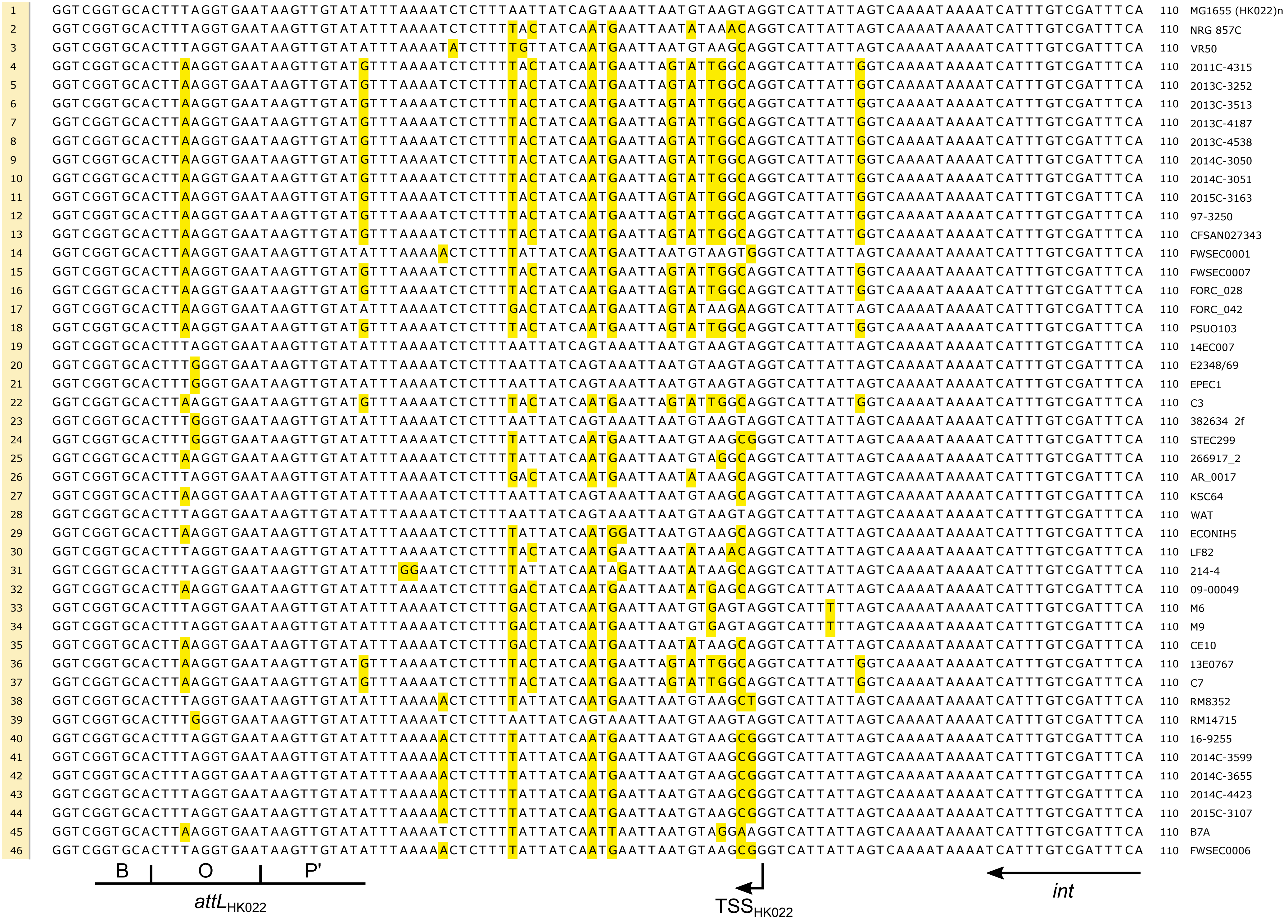
1 GGTCGGTGCACTTTAGGTGAATAAGTTGTATATTTAAAATCTCTTTAATTATCAGTAAATTAATGTAAGTAGGTCATTATTAGTCAAAATAAAATCATTTGTCGATTTCA 110 MG1655 (HK022)n
2 GGTCGGTGCACTTTAGGTGAATAAGTTGTATATTTAAAATCTCTTTTACTATCAATGAATTAATATAAACAGGTCATTATTAGTCAAAATAAAATCATTTGTCGATTTCA 110 NRG 857C
3 GGTCGGTGCACTTTAGGTGAATAAGTTGTATATTTAAAATATCTTTTGTTATCAATGAATTAATGTAAGCAGGTCATTATTAGTCAAAATAAAATCATTTGTCGATTTCA 110 VR50
4 GGTCGGTGCACTTAAGGTGAATAAGTTGTATGTTTAAAATCTCTTTTACTATCAATGAATTAGTATTGGCAGGTCATTATTGGTCAAAATAAAATCATTTGTCGATTTCA 110 2011C-4315
5 GGTCGGTGCACTTAAGGTGAATAAGTTGTATGTTTAAAATCTCTTTTACTATCAATGAATTAGTATTGGCAGGTCATTATTGGTCAAAATAAAATCATTTGTCGATTTCA 110 2013C-3252
6 GGTCGGTGCACTTAAGGTGAATAAGTTGTATGTTTAAAATCTCTTTTACTATCAATGAATTAGTATTGGCAGGTCATTATTGGTCAAAATAAAATCATTTGTCGATTTCA 110 2013C-3513
7 GGTCGGTGCACTTAAGGTGAATAAGTTGTATGTTTAAAATCTCTTTTACTATCAATGAATTAGTATTGGCAGGTCATTATTGGTCAAAATAAAATCATTTGTCGATTTCA 110 2013C-4187
8 GGTCGGTGCACTTAAGGTGAATAAGTTGTATGTTTAAAATCTCTTTTACTATCAATGAATTAGTATTGGCAGGTCATTATTGGTCAAAATAAAATCATTTGTCGATTTCA 110 2013C-4538
9 GGTCGGTGCACTTAAGGTGAATAAGTTGTATGTTTAAAATCTCTTTTACTATCAATGAATTAGTATTGGCAGGTCATTATTGGTCAAAATAAAATCATTTGTCGATTTCA 110 2014C-3050
10 GGTCGGTGCACTTAAGGTGAATAAGTTGTATGTTTAAAATCTCTTTTACTATCAATGAATTAGTATTGGCAGGTCATTATTGGTCAAAATAAAATCATTTGTCGATTTCA 110 2014C-3051
11 GGTCGGTGCACTTAAGGTGAATAAGTTGTATGTTTAAAATCTCTTTTACTATCAATGAATTAGTATTGGCAGGTCATTATTGGTCAAAATAAAATCATTTGTCGATTTCA 110 2015C-3163
12 GGTCGGTGCACTTAAGGTGAATAAGTTGTATGTTTAAAATCTCTTTTACTATCAATGAATTAGTATTGGCAGGTCATTATTGGTCAAAATAAAATCATTTGTCGATTTCA 110 97-3250
13 GGTCGGTGCACTTAAGGTGAATAAGTTGTATGTTTAAAATCTCTTTTACTATCAATGAATTAGTATTGGCAGGTCATTATTGGTCAAAATAAAATCATTTGTCGATTTCA 110 CFSAN027343
14 GGTCGGTGCACTTAAGGTGAATAAGTTGTATATTTAAAAACTCTTTTATTATCAATGAATTAATGTAAGTGGGTCATTATTAGTCAAAATAAAATCATTTGTCGATTTCA 110 FWSEC0001
15 GGTCGGTGCACTTAAGGTGAATAAGTTGTATGTTTAAAATCTCTTTTACTATCAATGAATTAGTATTGGCAGGTCATTATTGGTCAAAATAAAATCATTTGTCGATTTCA 110 FWSEC0007
16 GGTCGGTGCACTTAAGGTGAATAAGTTGTATGTTTAAAATCTCTTTTACTATCAATGAATTAGTATTGGCAGGTCATTATTGGTCAAAATAAAATCATTTGTCGATTTCA 110 FORC_028
17 GGTCGGTGCACTTAAGGTGAATAAGTTGTATATTTAAAATCTCTTTGACTATCAATGAATTAGTATAAGAAGGTCATTATTAGTCAAAATAAAATCATTTGTCGATTTCA 110 FORC_042
18 GGTCGGTGCACTTAAGGTGAATAAGTTGTATGTTTAAAATCTCTTTTACTATCAATGAATTAGTATTGGCAGGTCATTATTGGTCAAAATAAAATCATTTGTCGATTTCA 110 PSUO103
19 GGTCGGTGCACTTTAGGTGAATAAGTTGTATATTTAAAATCTCTTTAATTATCAGTAAATTAATGTAAGTAGGTCATTATTAGTCAAAATAAAATCATTTGTCGATTTCA 110 14EC007
20 GGTCGGTGCACTTTGGGTGAATAAGTTGTATATTTAAAATCTCTTTAATTATCAGTAAATTAATGTAAGTAGGTCATTATTAGTCAAAATAAAATCATTTGTCGATTTCA 110 E2348/69
21 GGTCGGTGCACTTTGGGTGAATAAGTTGTATATTTAAAATCTCTTTAATTATCAGTAAATTAATGTAAGTAGGTCATTATTAGTCAAAATAAAATCATTTGTCGATTTCA 110 EPEC1
22 GGTCGGTGCACTTAAGGTGAATAAGTTGTATGTTTAAAATCTCTTTTACTATCAATGAATTAGTATTGGCAGGTCATTATTGGTCAAAATAAAATCATTTGTCGATTTCA 110 C3
23 GGTCGGTGCACTTTGGGTGAATAAGTTGTATATTTAAAATCTCTTTAATTATCAGTAAATTAATGTAAGTAGGTCATTATTAGTCAAAATAAAATCATTTGTCGATTTCA 110 382634_2f
24 GGTCGGTGCACTTTGGGTGAATAAGTTGTATATTTAAAATCTCTTTTATTATCAATGAATTAATGTAAGCGGGTCATTATTAGTCAAAATAAAATCATTTGTCGATTTCA 110 STEC299
25 GGTCGGTGCACTTAAGGTGAATAAGTTGTATATTTAAAATCTCTTTTATTATCAATGAATTAATGTAGGCAGGTCATTATTAGTCAAAATAAAATCATTTGTCGATTTCA 110 266917_2
26 GGTCGGTGCACTTTAGGTGAATAAGTTGTATATTTAAAATCTCTTTGACTATCAATGAATTAATATAAGCAGGTCATTATTAGTCAAAATAAAATCATTTGTCGATTTCA 110 AR_0017
27 GGTCGGTGCACTTAAGGTGAATAAGTTGTATATTTAAAATCTCTTTAATTATCAGTAAATTAATGTAAGCAGGTCATTATTAGTCAAAATAAAATCATTTGTCGATTTCA 110 KSC64
28 GGTCGGTGCACTTTAGGTGAATAAGTTGTATATTTAAAATCTCTTTAATTATCAGTAAATTAATGTAAGTAGGTCATTATTAGTCAAAATAAAATCATTTGTCGATTTCA 110 WAT
29 GGTCGGTGCACTTAAGGTGAATAAGTTGTATATTTAAAATCTCTTTTATTATCAATGGATTAATGTAAGCAGGTCATTATTAGTCAAAATAAAATCATTTGTCGATTTCA 110 ECONIH5
30 GGTCGGTGCACTTTAGGTGAATAAGTTGTATATTTAAAATCTCTTTTACTATCAATGAATTAATATAAACAGGTCATTATTAGTCAAAATAAAATCATTTGTCGATTTCA 110 LF82
31 GGTCGGTGCACTTTAGGTGAATAAGTTGTATATTTGGAATCTCTTTTATTATCAATAGATTAATATAAGCAGGTCATTATTAGTCAAAATAAAATCATTTGTCGATTTCA 110 214-4
32 GGTCGGTGCACTTAAGGTGAATAAGTTGTATATTTAAAATCTCTTTGACTATCAATGAATTAATATGAGCAGGTCATTATTAGTCAAAATAAAATCATTTGTCGATTTCA 110 09-00049
33 GGTCGGTGCACTTTAGGTGAATAAGTTGTATATTTAAAATCTCTTTGACTATCAATGAATTAATGTGAGTAGGTCATTTTTAGTCAAAATAAAATCATTTGTCGATTTCA 110 M6
34 GGTCGGTGCACTTTAGGTGAATAAGTTGTATATTTAAAATCTCTTTGACTATCAATGAATTAATGTGAGTAGGTCATTTTTAGTCAAAATAAAATCATTTGTCGATTTCA 110 M9
35 GGTCGGTGCACTTAAGGTGAATAAGTTGTATATTTAAAATCTCTTTGACTATCAATGAATTAATATAAGCAGGTCATTATTAGTCAAAATAAAATCATTTGTCGATTTCA 110 CE10
36 GGTCGGTGCACTTAAGGTGAATAAGTTGTATGTTTAAAATCTCTTTTACTATCAATGAATTAGTATTGGCAGGTCATTATTGGTCAAAATAAAATCATTTGTCGATTTCA 110 13E0767
37 GGTCGGTGCACTTAAGGTGAATAAGTTGTATGTTTAAAATCTCTTTTACTATCAATGAATTAGTATTGGCAGGTCATTATTGGTCAAAATAAAATCATTTGTCGATTTCA 110 C7
38 GGTCGGTGCACTTTAGGTGAATAAGTTGTATATTTAAAAACTCTTTTATTATCAATGAATTAATGTAAGCTGGTCATTATTAGTCAAAATAAAATCATTTGTCGATTTCA 110 RM8352
39 GGTCGGTGCACTTTGGGTGAATAAGTTGTATATTTAAAATCTCTTTAATTATCAGTAAATTAATGTAAGTAGGTCATTATTAGTCAAAATAAAATCATTTGTCGATTTCA 110 RM14715
40 GGTCGGTGCACTTTAGGTGAATAAGTTGTATATTTAAAAACTCTTTTATTATCAATGAATTAATGTAAGCGGGTCATTATTAGTCAAAATAAAATCATTTGTCGATTTCA 110 16-9255
41 GGTCGGTGCACTTTAGGTGAATAAGTTGTATATTTAAAAACTCTTTTATTATCAATGAATTAATGTAAGCGGGTCATTATTAGTCAAAATAAAATCATTTGTCGATTTCA 110 2014C-3599
42 GGTCGGTGCACTTTAGGTGAATAAGTTGTATATTTAAAAACTCTTTTATTATCAATGAATTAATGTAAGCGGGTCATTATTAGTCAAAATAAAATCATTTGTCGATTTCA 110 2014C-3655
43 GGTCGGTGCACTTTAGGTGAATAAGTTGTATATTTAAAAACTCTTTTATTATCAATGAATTAATGTAAGCGGGTCATTATTAGTCAAAATAAAATCATTTGTCGATTTCA 110 2014C-4423
44 GGTCGGTGCACTTTAGGTGAATAAGTTGTATATTTAAAAACTCTTTTATTATCAATGAATTAATGTAAGCGGGTCATTATTAGTCAAAATAAAATCATTTGTCGATTTCA 110 2015C-3107
45 GGTCGGTGCACTTAAGGTGAATAAGTTGTATATTTAAAATCTCTTTTATTATCAATTAATTAATGTAGGAAGGTCATTATTAGTCAAAATAAAATCATTTGTCGATTTCA 110 B7A
46 GGTCGGTGCACTTTAGGTGAATAAGTTGTATATTTAAAAACTCTTTTATTATCAATGAATTAATGTAAGCGGGTCATTATTAGTCAAAATAAAATCATTTGTCGATTTCA 110 FWSEC0006
B
O
P'
attL HK022
TSS HK022
int

1	ATTTTGTCCCATTCCCGTCCACGGCTGTCCCTATACCGCGCCGCCATTGAATCTGATTTATGCCCGAGAAGACGTTGAGCAAACTTATCGCCAATCTGGTTCCGGTATAG	220	MG1655 (HK022)n
2	ATTTTGTCCCATTCACGCCCACGACTGTCCCTATACCGCGCCGCCATTGAATCTGATTTATGCCCGAGAAGACGTTGAGCAAACTTATCGCCAATCTGATTCCGGTATAG	220	NRG 857C
3	ATTTTGTCCCATTCCCGTCCACGGCTGTCCCTATACCGCGCCGCCATTGAATCTGATTTATGCCCGAGAAGACGTTGAGCAAACTTATCGCCAATCTGGTTCCGGTATAG	220	VR50
4	ATTTTGTCCCATTCCCGTCCACGGCTGTCTCTATACCGCGCCGCCATTGAATCTGATTTATGCCCGAGAAGACGTTGAGCAAATTTATCGCCAATCTGGTTCCGGTATAG	220	2011C-4315
5	ATTTTGTCCCATTCCCGTCCACGGCTGTCTCTATACCGCGCCGCCATTGAATCTGATTTATGCCCGAGAAGACGTTGAGCAAATTTATCGCCAATCTGGTTCCGGTATAG	220	2013C-3252
6	ATTTTGTCCCATTCCCGTCCACGGCTGTCTCTATACCGCGCCGCCATTGAATCTGATTTATGCCCGAGAAGACGTTGAGCAAATTTATCGCCAATCTGGTTCCGGTATAG	220	2013C-3513
7	ATTTTGTCCCATTCCCGTCCACGGCTGTCTCTATACCGCGCCGCCATTGAATCTGATTTATGCCCGAGAAGACGTTGAGCAAATTTATCGCCAATCTGGTTCCGGTATAG	220	2013C-4187
8	ATTTTGTCCCATTCCCGTCCACGGCTGTCTCTATACCGCGCCGCCATTGAATCTGATTTATGCCCGAGAAGACGTTGAGCAAATTTATCGCCAATCTGGTTCCGGTATAG	220	2013C-4538
9	ATTTTGTCCCATTCCCGTCCACGGCTGTCTCTATACCGCGCCGCCATTGAATCTGATTTATGCCCGAGAAGACGTTGAGCAAATTTATCGCCAATCTGGTTCCGGTATAG	220	2014C-3050
10	ATTTTGTCCCATTCCCGTCCACGGCTGTCTCTATACCGCGCCGCCATTGAATCTGATTTATGCCCGAGAAGACGTTGAGCAAATTTATCGCCAATCTGGTTCCGGTATAG	220	2014C-3051
11	ATTTTGTCCCATTCCCGTCCACGGCTGTCTCTATACCGCGCCGCCATTGAATCTGATTTATGCCCGAGAAGACGTTGAGCAAATTTATCGCCAATCTGGTTCCGGTATAG	220	2015C-3163
12	ATTTTGTCCCATTCCCGTCCACGGCTGTCTCTATACCGCGCCGCCATTGAATCTGATTTATGCCCGAGAAGACGTTGAGCAAACTTATCGCCAATCTGATTCCGGTATAG	220	97-3250
13	ATTTTGTCCCATTCCCGTCCACGGCTGTCTCTATACCGCGCCGCCATTGAATCTGATTTATGCCCGAGAAGACGTTGAGCAAATTTATCGCCAATCTGGTTCCGGTATAG	220	CFSAN027343
14	ATTTTGTCCCATTCCCGTCCACGGCTGTCCCTATACCGCGCCGCCATTAAATCTGATTTATGCCCGAGAAGACGTTGAGCAAACTTATCGCCAATCTGGTTCCGGTATAG	220	FWSEC0001
15	ATTTTGTCCCATTCCCGTCCACGGCTGTCTCTATACCGCGCCGCCATTGAATCTGATTTATGCCCGAGAAGACGTTGAGCAAATTTATCGCCAATCTGGTTCCGGTATAG	220	FWSEC0007
16	ATTTTGTCCCATTCCCGTCCACGGCTGTCTCTATACCGCGCCGCCATTGAATCTGATTTATGCCCGAGAAGACGTTGAGCAAACTTATCGCCAATCTGATTCCGGTATAG	220	FORC_028
17	ATTTTGTCCCATTCCCGTCCACGGCTGTCCCTATACCGCGCCGCCATTGAATCTGATTTATGCCCGAGAAGGCGTTGAGCAAACTTATCGCCAATCTGGTTCCGGTATAG	220	FORC_042
18	ATTTTGTCCCATTCCCGTCCACGGCTGTCTCTATACCGCGCCGCCATTGAATCTGATTTATGCCCGAGAAGACGTTGAGCAAATTTATCGCCAATCTGGTTCCGGTATAG	220	PSUO103
19	ATTTTGTCCCATTCCCGTCCACGGCTGTCCCTATACCGCGCCGCCATTGAGTCTGATTTATGCCCGAGAAGACGTTGAGCAAACTTATCGCCAATCTGGTTCCGGTATAG	220	14EC007
20	ATTTTGTCCCATTCCCGTCCACGGCTGTCCCTATACCGCGCCGCCATTGAATCTGATTTATGCCCGAGAAGACGTTGAGCAAACTTATCGCCAATCTGGTTCCGGTATAG	220	E2348/69
21	ATTTTGTCCCATTCCCGTCCACGGCTGTCCCTATACCGCGCCGCCATTGAATCTGATTTATGCCCGAGAAGACGTTGAGCAAACTTATCGCCAATCTGGTTCCGGTATAG	220	EPEC1
22	ATTTTGTCCCATTCCCGTCCACGGCTGTCTCTATACCGCGCCGCCATTGAATCTGATTTATGCCCGAGAAGACGTTGAGCAAATTTATCGCCAATCTGGTTCCGGTATAG	220	C3
23	ATTTTGTCCCATTCCCGTCCACGGCTGTCCCTATACCGCGCCGCCATTGAATCTGATTTATGCCCGAGAAGACGTTGAGCAAACTTATCGCCAATCTGGTTCCGGTATAG	220	382634_2f
24	ATTTTGTCCCATTCCCGTCCACGGCTATCCCTATACCGCGCAGCCATTGAATCTGATTTATGCCCGAGAAGACGTTGAGCAAACTTATCGCCAATCTGGTTCCGGTATAG	220	STEC299
25	ATTTTGTCCCATTCCCGTCCACGGCTGTCCCTATACCGCGCCGCCATTGAATCTGATTTATGCCCGAGAAGACGTTGAGCAAACTTATCGCCAATCTGGTTCCGGTATAG	220	266917_2
26	ATTTTGTCCCATTCCCGTCCACGGCTATCCCTATACCGCGCAGCCATTGAATCTGATTTATGCCCGAGAAGACGTTGAGCAAACTTATCGCCAATCTGGTTCCGGTATAG	220	AR_0017
27	ATTTTGTCCCATTCCCGTCCACGGCTGTCCCTATACCGCGCCGCCATTGAATCTGATTTATGCCCGAGAAGACGTTGAGCAAACTTATCGCCAATCTGGTTCCGGTATAG	220	KSC64
28	ATTTTGTCCCATTCCCGTCCACGGCTGTCCCTATACCGCGCCGCCATTGAATCTGATTTATGCCCGAGAAGACGTTGAGCAAACTTATCGCCAATCTGGTTCCGGTATAG	220	WAT
29	ATTTTGTCCCATTCCCGTCCACGGCTGTCCCTATACCGCGCCGCCATTGAATCTGATTTATGTCCGAGAAGACGTTGAGCAAACTTATCGCCAATCTGGTTCCGGTATAG	220	ECONIH5
30	ATTTTGTCCCATTCACGCCCACGACTGTCCCTATACCGCGCCGCCATTGAATCTGATTTATGCCCGAGAAGACGTTGAGCAAACTTATCGCCAATCTGATTCCGGTATAG	220	LF82
31	ATTTTGTCCCATTCCCGTCCACGGCTGTCCCTATACCGCGCCGCCATTGAATCTGATTTATGCCCGAGAAGACGTTGAGCAAACTTATCGCCAATCTGGTTCCGGTATAG	220	214-4
32	ATTTTGTCCCATTCCCGTCCACGGCTGTCCCTGTACCGTGCCGCCATTGAATCTGATTTATGCCCGAGAAGACGTTGAGCAAACTTATCGCCAATCTGGTTCCGGTATAG	220	09-00049
33	ATTTTGTCCCATTCCCGTCCACGGCTGTCCCTATACCGCGCCGCCATTGAATCTGATTTATGCCCGAGAAGACGTTGAGCAAACTTATCGCCAATCTGGTTCCGGTATAG	220	M6
34	ATTTTGTCCCATTCCCGTCCACGGCTGTCCCTATACCGCGCCGCCATTGAATCTGATTTATGCCCGAGAAGACGTTGAGCAAACTTATCGCCAATCTGGTTCCGGTATAG	220	M9
35	ATTTTGTCCCATTCCCGTCCACGGCTATCCCTATACCGCGCAGCCATTGAATCTGATTTATGCCCGAGAAGACGTTGAGCAAACTTATCGCCAATCTGGTTCCGGTATAG	220	CE10
36	ATTTTGTCCCATTCCCGTCCACGGCTGTCTCTATACCGCGCCGCCATTGAATCTGATTTATGCCCGAGAAGACGTTGAGCAAATTTATCGCCAATCTGGTTCCGGTATAG	220	13E0767
37	ATTTTGTCCCATTCCCGTCCACGGCTGTCTCTATACCGCGCCGCCATTGAATCTGATTTATGCCCGAGAAGACGTTGAGCAAATTTATCGCCAATCTGGTTCCGGTATAG	220	C7
38	ATTTTGTCCCATTCCCGTCCACGGCTGTCCCTATACCGCGCCGCCATTAAATCTGATTTATGCCCGAGAAGACGTTGAGCAAACTTATCGCCAATCTGGTTCCGGTATAG	220	RM8352
39	ATTTTGTCCCATTCCCGTCCACGGCTGTCCCTATACCGCGCCGCCATTGAGTCTGATTTATGCCCGAGAAGACGTTGAGCAAACTTATCGCCAATCTGGTTCCGGTATAG	220	RM14715
40	ATTTTGTCCCATTCCCGTCCACGGCTGTCCCTATACCGCGCCGCCATTAAATCTGATTTATGCCCGAGAAGACGTTGAGCAAACTTATCGCCAATCTGGTTCCGGTATAG	220	16-9255
41	ATTTTGTCCCATTCCCGTCCACGGCTGTCCCTATACCGCGCCGCCATTAAATCTGATTTATGCCCGAGAAGACGTTGAGCAAACTTATCGCCAATCTGGTTCCGGTATAG	220	2014C-3599
42	ATTTTGTCCCATTCCCGTCCACGGCTGTCCCTATACCGCGCCGCCATTAAATCTGATTTATGCCCGAGAAGACGTTGAGCAAACTTATCGCCAATCTGGTTCCGGTATAG	220	2014C-3655
43	ATTTTGTCCCATTCCCGTCCACGGCTGTCCCTATACCGCGCCGCCATTAAATCTGATTTATGCCCGAGAAGACGTTGAGCAAACTTATCGCCAATCTGGTTCCGGTATAG	220	2014C-4423
44	ATTTTGTCCCATTCCCGTCCACGGCTGTCCCTATACCGCGCCGCCATTAAATCTGATTTATGCCCGAGAAGACGTTGAGCAAACTTATCGCCAATCTGGTTCCGGTATAG	220	2015C-3107
45	ATTTTGTCCCATTCCCGTCCACGGCTGTCCCTATACCGCGCCGCCATTGAGTCTGATTTATGCCCGAGAAGACGTTGAGCAAACTTATCGCCAATCTGGTTCCGGTATAG	220	B7A
46	ATTTTGTCCCATTCCCGTCCACGGCTGTCCCTATACCGCGCCGCCATTAAATCTGATTTATGCCCGAGAAGACGTTGAGCAAACTTATCGCCAATCTGGTTCCGGTATAG	220	FWSEC0006

int
