# Supplementary material for: Phage integration alters the respiratory strategy of its host
Source: eLife. 2019 Oct 25;8:e49081. doi: 10.7554/eLife.49081 (PMC6814406; doi:10.7554/eLife.49081)
Supplement: Supplementary file 3. [file elife-49081-supp3.docx]

| **Strain** | **Relevant genotype** | **Reference / source** |
| --- | --- | --- |
| MG1655 |  | Coli Genetic Stock Center #7740, Blattner et al., 1997 |
| DFE12 | MG1655 *attB*_λ_::(*cat* P*_torCAD_-yfp*) *ompA-cfp* (HK022)_n_ | This work |
| DFE33 | SASX41B Δ*lacIZY*::P*_torCAD_-yfp*-FRT-*kan*-FRT | This work |
| DFE34 | NRG 857C Δ*lacIZY*::P*_torCAD_-yfp*-FRT-*kan*-FRT | This work |
| JNC151 | MG1655 (HK022)_n_ | This work |
| JNC163 | MG1655 Δ*lacZYA*::FRT-*cat*-FRT *torT-lacZ*-FRT-*kan*-FRT Δ*torR* | Carey et al., 2018 |
| JNC166 | MG1655 Δ*lacZYA*::FRT *torS-lacZ*-FRT-*kan*-FRT | Carey et al., 2018 |
| JNC168 | MG1655 Δ*lacZYA*::FRT-*cat*-FRT (HK022)_n_ *torT-lacZ*-FRT-*kan*-FRT Δ*torR* | This work |
| JNC169 | MG1655 Δ*lacZYA*::FRT *torS-lacZ*-FRT-*kan*-FRT (HK022)_n_ | This work |
| JNC173 | MG1655 Δ*fhuA*::FRT-*kan*-FRT *attB*_λ_::(*cat* P*_torCAD_-yfp*) *ompA-cfp* (HK022)_n_ | This work |
| JNC174 | MG1655 Δ*fhuA*::FRT-*kan*-FRT *attB*_λ_::(*cat* P*_torCAD_-yfp*) Δ*xylAFG*::P*_tetA_*-*mcherry*-FRT | This work |
| JNC175 | MG1655 Δ*lacZYA*::FRT *torS-lacZ*-FRT-*kan*-FRT (HK022)_n_ *attL*_HK022_::*Ω* | This work |
| JW0146 | F^-^ λ^-^ Δ*araBAD* Δ*fhuA*::FRT-*kan*-FRT Δ*lacZ rph-1* Δ*rhaBAD hsdR514* | Baba et al., 2006 |
| MMR8 | MG1655 *attB*_λ_::(*cat* P*_torCAD_-yfp*) *ompA-cfp* | Roggiani and Goulian, 2015 |
| MMR129 | MG1655 Δ*lacIZY*::P*_torCAD_-yfp*-FRT-*kan*-FRT | Goulian lab stock |
| NRG 857C |  | Nash et al., 2010 |
| PK4854 | MG1655 Δ*iscR*::FRT | Schwartz et al., 2001 |
| PK12556 | pKD46 in MG1655 | This work |
| PK13196 | MG1655 *lacZ*::*kan*-P*_torS_*-(GTG)*lacZ* Δ*iscR*::FRT | This work |
| PK13199 | MG1655 *lacZ*::*kan*-P*_torS_*-(ATG)*lacZ* Δ*iscR*::FRT | This work |
| SASX41B | Hfr(PO2A) *hemA41 relA1 spoT1 metB1 rrnB-2 mcrB1 creC510* | Coli Genetic Stock Center #4806 |

**References**

Baba T, Ara T, Hasegawa M, Takai Y, Okumura Y, Baba M, Datsenko KA, Tomita M, Wanner BL, Mori H. 2006. Construction of *Escherichia coli* K-12 in-frame, single-gene knockout mutants: the Keio collection. *Molecular Systems Biology* **2**:2006.0008. DOI: https://doi.org/10.1038/msb4100050, PMID: 16738554

Blattner FR, Plunkett G, Bloch CA, Perna NT, Burland V, Riley M, Collado-Vides J, Glasner JD, Rode CK, Mayhew GF, Gregor J, Davis NW, Kirkpatrick HA, Goeden MA, Rose DJ, Mau B, Shao Y. 1997. The complete genome sequence of *Escherichia coli* K-12. *Science* **277**:1453–1462. DOI: https://doi.org/10.1126/science.277.5331.1453, PMID: 9278503

Carey JN, Mettert EL, Roggiani M, Myers KS, Kiley PJ, Goulian M. 2018. Regulated stochasticity in a bacterial signaling network permits tolerance to a rapid environmental change. *Cell* **175**:1989–1990. DOI: https://doi.org/10.1016/j.cell.2018.11.051, PMID: 30550792

Nash JH, Villegas A, Kropinski AM, Aguilar-Valenzuela R, Konczy P, Mascarenhas M, Ziebell K, Torres AG, Karmali MA, Coombes BK. 2010. Genome sequence of adherent-invasive *Escherichia coli* and comparative genomic analysis with other *E. coli* pathotypes. *BMC Genomics* **11**:667. DOI: https://doi.org/10.1186/1471-2164-11-667, PMID: 21108814

Roggiani M, Goulian M. 2015. Oxygen-dependent cell-to-cell variability in the output of the *Escherichia coli* Tor phosphorelay. *Journal of Bacteriology* **197**:1976–1987. DOI: https://doi.org/10.1128/JB.00074-15, PMID: 25825431

Schwartz CJ, Giel JL, Patschkowski T, Luther C, Ruzicka FJ, Beinert H, Kiley PJ. 2001. IscR, an Fe-S cluster-containing transcription factor, represses expression of *Escherichia coli* genes encoding Fe-S cluster assembly proteins. *PNAS* **98**:14895–14900. DOI: https://doi.org/10.1073/pnas.251550898, PMID: 11742080
