# Supplementary material for: Phage integration alters the respiratory strategy of its host
Source: eLife. 2019 Oct 25;8:e49081. doi: 10.7554/eLife.49081 (PMC6814406; doi:10.7554/eLife.49081)
Supplement: Supplementary file 4. [file elife-49081-supp4.docx]

| **Plasmid** | **Relevant genotype** | **Reference / source** |
| --- | --- | --- |
| pDSW206 | ori(pBR322) *lacI*^q^ *amp* P*_trc_* attenuated promoter | Weiss et al., 1999 |
| pJB31 | ori(RSF1010) *Ω strA strB* | Beaupré et al., 1997 |
| pKD46 | ori(pSC101) *rep101*(ts) P*_araBAD_-gam-bet-exo araC amp* | Datsenko and Wanner, 2000 |
| pMR26 | pDSW206 *torT* | Roggiani and Goulian, 2015 |
| pPK7035 | ori(pBR322) *amp* *kan lacZ’* | Kang et al., 2005 |
| pPK7179 | ori(pBR322) ter(*spf*) *amp RNA-1* | Kang et al., 2005 |
| pPK12669 | pPK7179 with -152 to +28 bp relative to the *torS* ATG start codon from MG1655 in XhoI/BamHI sites | This work |
| pPK12792 | pPK7035 with -152 to +28 bp relative to the *torS* ATG start codon in XhoI/BamHI sites | This work |
| pPK13169 | pPK12792 but with *kan*-P*_torS_*-(GTG)*lacZ’* | This work |
| pPK13171 | pPK12792 but with *kan*-P*_torS_*-(ATG)*lacZ’* | This work |
| pPK13256 | pPK7179 with -231 to +28 bp relative to the *torS* ATG start codon from JNC151 in XhoI/BamHI sites | This work |

**References**

Beaupré CE, Bohne J, Dale EM, Binns AN. 1997. Interactions between VirB9 and VirB10 membrane proteins involved in movement of DNA from *Agrobacterium tumefaciens* into plant cells. *Journal of Bacteriology* **179**: 78–89. DOI: https://doi.org/10.1128/jb.179.1.78-89.1997, PMID: 8981983

Datsenko KA, Wanner BL. 2000. One-step inactivation of chromosomal genes in *Escherichia coli* K-12 using PCR products. *PNAS* **97**:6640–6645. DOI: https://doi.org/10.1073/pnas.120163297, PMID: 10829079

Kang Y, Weber KD, Qiu Y, Kiley PJ, Blattner FR. 2005. Genome-wide expression analysis indicates that FNR of *Escherichia coli* K-12 regulates a large number of genes of unknown function. *Journal of Bacteriology* **187**: 1135–1160. DOI: https://doi.org/10.1128/JB.187.3.1135-1160.2005, PMID: 15659690

Roggiani M, Goulian M. 2015. Oxygen-dependent cell-to-cell variability in the output of the *Escherichia coli* Tor phosphorelay. *Journal of Bacteriology* **197**:1976–1987. DOI: https://doi.org/10.1128/JB.00074-15, PMID: 25825431

Weiss DS, Chen JC, Ghigo JM, Boyd D, Beckwith J. 1999. Localization of FtsI (PBP3) to the septal ring requires its membrane anchor, the Z ring, FtsA, FtsQ, and FtsL. *Journal of Bacteriology* **181**:508–520. PMID: 9882665
